# Extracellular matrix stiffness regulates the proliferation and migration capacities of lymphatic endothelial cells via FAT1

**DOI:** 10.3389/fcell.2025.1667154

**Published:** 2025-10-27

**Authors:** Zhangrun Xu, Jian Yu, Dandan Chen, Yiwen Liu, Guoliang Zhang, Qian Guo, Cuixia Yang, Feng Gao, Yiqing He, Yan Du

**Affiliations:** ^1^ Department of Molecular Biology, Shanghai Sixth People’s Hospital Affiliated to Shanghai Jiao Tong University School of Medicine, Shanghai, China; ^2^ Department of Clinical Laboratory, Shanghai Sixth People’s Hospital Affiliated to Shanghai Jiao Tong University School of Medicine, Shanghai, China; ^3^ Department of Transfusion Medicine, Shanghai Sixth People’s Hospital Affiliated to Shanghai Jiao Tong University School of Medicine, Shanghai, China

**Keywords:** matrix stiffness, lymphatic endothelial cells, Fat1, focal adhesion, Wnt pathway

## Abstract

**Introduction:**

The extracellular matrix (ECM) stiffness serves as a critical biomechanical regulator of cellular behavior. However, its specific roles on lymphatic endothelial cells (LECs) remains poorly characterized, particularly in the context of tumorigenesis where progressive matrix stiffening is a hallmark of the tumor microenvironments (TME).

**Methods:**

The effects of ECM stiffness on LEC proliferation and migration were assessed using a tunable polyacrylamide hydrogel system. Differential gene expression in LECs on soft versus stiff substrates was identified by RNA-seq. To evaluate the stiffness-dependent regulation of FAT1 and its downstream mechanisms, we performed RT-qPCR, Western blot, immunofluorescence, wound healing, and spheroid assays. Furthermore, immunofluorescence was also used to compare FAT1 expression in tumor-associated versus normal lymphatic vessels.

**Results:**

In this study, we demonstrated that ECM stiffening significantly promotes LEC proliferation and migration. Notably, we observed marked downregulation of FAT1 expression in LECs cultured on tumor stiffness-mimicking matrix, a finding validated in clinical breast cancer specimens and murine models of breast cancer and melanoma. Mechanistic investigations identified FAT1 as a pivotal mechanotransducer that orchestrates LECs functional responses to biomechanical cues. Specifically, the knockdown of FAT1 facilitated β-catenin nuclear translocation, activating transcription of cell cycle regulators Myc and Cyclin D1 to coordinately promote LEC proliferation. Furthermore, FAT1 deficiency increased LEC mechanosensitivity by modulating focal adhesion formation, inducing cytoskeleton reorganization and consequent enhancement of migratory potentials.

**Discussion:**

Together, our study uncovers FAT1 as a pivotal mechanosensor in LECs and highlight its significance in the biomechanical regulation. Targeting the FAT1-mediated signaling pathways may serve as a novel therapeutic strategy to inhibit tumor lymphatic metastasis.

## 1 Introduction

Mounting evidence from cancer mechanobiology research has established that solid tumors, particularly breast carcinomas, exhibit significantly increased tissue stiffness relative to normal physiological tissues (Jiang et al., 2022). This pathological matrix stiffening has emerged as a defining biomechanical characteristic of the tumor microenvironment (TME), driving malignant progression through multiple interconnected mechanisms: (1) activation of epithelial-mesenchymal transition programs, (2) enhancement of tumor cell invasiveness and metastatic potential, and (3) maintenance of cancer stem cell properties (Fattet et al., 2020; Reid et al., 2017; Liu et al., 2023; Chaudhuri et al., 2014). Moreover, increased matrix stiffness affects not only malignant cells but also the surrounding stromal compartment. Blood endothelial cells exhibit remarkable mechanosensitivity, developing multiple pathological phenotypes when exposed to stiff substrates, including aberrant angiogenic patterning (Bordeleau et al., 2016), metabolic reprogramming (Bertero et al., 2016), glycocalyx destabilization (Mahmoud et al., 2021), loss of endothelia identity markers (Zhang et al., 2015), and initiation of endothelial-to-mesenchymal transition (Zamani et al., 2022). These collective findings establish matrix mechanics as a fundamental regulator of diverse cellular processes within the TME.

Lymphatic endothelial cells (LECs), which are anatomically distinct from vascular endothelial cells due to their discontinuous basement membrane (Oliver et al., 2020), maybe particularly susceptible to extracellular matrix (ECM)-derived mechanical cues. While substantial research has focused on biochemical regulation of LECs by TME-secreted cytokines, our previous work identified tumor-derived ECM degradation products as potent mediators of LEC proliferation, migration (Jiang et al., 2025), and junctional integrity disruption (Du et al., 2016). However, the biomechanical regulation of lymphatic function remains poorly understood.

Current understanding suggests that soft matrix environments help maintain LEC phenotype, support lymphatic tube formation (Alderfer et al., 2021; Qin et al., 2024), and significantly influences early lymphatic morphogenesis (Frye et al., 2018). Although preliminary clinical evidence suggests a correlation between increased matrix stiffness and lymphangiogenesis in breast cancer (Cha et al., 2016), the precise molecular mechanisms underlying LEC mechanotransduction in stiff TME remain elusive.

This study aims to systematically investigate how matrix stiffening regulates LEC function from a biomechanical perspective, with particular emphasis on: 1. Characterizing stiffness-dependent alterations in LEC phenotype and function. 2. Identifying key molecular mediators of LEC mechanotransduction. 3. Elucidating downstream signaling pathways involved in matrix stiffness-induced lymphatic remodeling.

## 2 Materials and methods

### 2.1 Cell culture

Mouse lymphatic endothelial cells SVEC4-10 (mLECs) were obtained from the American Type Culture Collection. Mouse breast cancer cell 4T1 cells were purchased from the Cell Bank of the Type Culture Collection of the Chinese Academy of Sciences (Shanghai, China). Human lymphatic endothelial cells (hLECs) were obtained from the PromoCell (C-12216, Heidelberg, Germany). Mouse melanoma cancer cell B16F10 cells were purchased from the Cell Bank of the Type Culture Collection of the Chinese Academy of Sciences (Shanghai, China). MS1 mouse endothelial cells were obtained from the Cell Bank of the Type Culture Collection of the Chinese Academy of Sciences (Shanghai, China). SVEC4-10, B16F10, and MS1 cells were cultured in Dulbecco’s modified Eagle’s medium (BasalMedia, Shanghai, China) respectively supplemented with 10% FBS (BasalMedia, Shanghai, China), 100 U/mL penicillin and 100 mg/mL streptomycin. Human lymphatic endothelial cells were cultured in EGM-2 medium (CC-3202, Lonza, BSL, Switzerland), and cells within passage numbers 4 to 7 were used for all experiments. 4T1 cells were cultured in RPMI 1640 medium (BasalMedia, Shanghai, China) respectively supplemented with 10% FBS (BasalMedia, Shanghai, China), 100 U/mL penicillin and 100 mg/mL streptomycin. All cells were tested for *mycoplasma* and were mycoplasma-negative. All cells were cultured in a humidified atmosphere under 5% CO_2_.

### 2.2 Preparation of polyacrylamide hydrogels

Polyacrylamide (PA) hydrogels with mechanically tunable stiffness were prepared as previously described (Tse and Engler, 2010). Briefly, coverslips were soaked in 3-aminopropyltriethoxysilane (ST1087, Beyotime) at room temperature for 10 min. The coverslips were then thoroughly washed with sterile water and immersed in 0.5% glutaraldehyde (G849973, Macklin) for 30 min, followed by another extensive wash in sterile water and air drying for later use. Glass slides were immersed in dimethyldichlorosilane (D806824, Macklin) for 5 min. PA hydrogels with soft and stiff stiffness were prepared by adjusting the relative concentrations of acrylamide and bis-acrylamide. Different concentrations of 40% acrylamide (A6299, Macklin) and 2% bis-acrylamide (M6024, Macklin), 1:100 (v/v) ammonium persulfate (AR1166, Boster), and 1:1,000 (v/v) TEMED (AR1165, Boster) were mixed for polymerization. The gel mixture was quickly pipetted onto the slides, and treated coverslips (treated side down) were carefully placed onto the gel droplets. The gels were allowed to polymerize for 1 h. After polymerization, the bottom glass slide was removed, and the top coverslip-gel composite was transferred to a tissue culture plate and washed with 50 mM HEPES (C0215, Beyotime) to remove unpolymerized gel. To promote cell adhesion, the surface of the hydrogel was functionalized by applying sulfosuccinimidyl-6-(4-azido-2-nitrophenylamino)-hexanoate (sulfo-SANPAH, ProteoChem) and irradiating under 365-nm UV light (10W) for 6 min. The gels were then washed with 50 mM HEPES buffer and incubated with 0.1 mg/mL rat-tail collagen I (354,236, Corning) solution at 4 °C overnight. Finally, the coverslips were rinsed twice with PBS and sterilized under UV light for 30 min before cell seeding.

### 2.3 Patients and animal tissue samples

This study was approved by the Ethics Committee of Shanghai Jiao Tong University Affiliated Sixth People’s Hospital. Patients’ privacy rights of human subjects have been observed and written informed consent was obtained from all patients. Normal and tumor breast tissue samples were collected from patients who underwent mastectomy at Shanghai Sixth People’s Hospital. Patients who had received chemotherapy or radiotherapy prior to surgery were excluded.

For animal studies, two distinct tumor models were established. In the first model, 5 × 10^5^ 4T1 cells in 100 μL PBS were injected into the left fourth mammary fat pad of randomly selected 6–8-week-old female BALB/c mice. Control mice were injected with PBS alone. Ten days later, when the primary tumor diameter reached 1 cm, tumor tissues in the 4T1 group and mammary fat pads in the PBS group were collected for further analysis. Parallelly, 3 × 10^6^ B16F10 melanoma cells in 25 μL PBS were implanted subcutaneously into the footpads of 6–8-week-old C57BL/6 J mice. Control mice were injected with PBS alone. Ten days later, tumor tissues in the B16F10 group and footpads in the PBS group were collected for further analysis. All animal experiments were approved by the Animal Care and Use Committee.

### 2.4 Cell immunofluorescence and analysis

Cells seeded on hydrogels with different stiffness were fixed in 4% paraformaldehyde (PFA) for 15 min, permeabilized with 0.1% Triton X-100 for 10 min at room temperature, and blocked with 3% bovine serum albumin (BSA) for 1 h. Cells were then incubated overnight at 4 °C with primary antibodies against Ki67 (14–5,698, 1:300, Invitrogen), FAT1 (ab190242, 1:200, Abcam), YAP (sc-101199, 1:300, Santa Cruz), Vinculin (V9131, 1:300, Sigma), and β-catenin (ab32572, 1:200, Abcam), non-phospho (active) β-catenin (8,814, 1:200, Cell Signaling Technology). After washing, cells were incubated with Alexa Fluor 594- or 488-conjugated secondary antibodies (Abcam, 1:1,000) for 1 h at room temperature. F-actin was stained with DyLight 647 phalloidin (40762ES75, Yeasen), and nuclei were counterstained with DAPI (D9542, Sigma). Images were acquired using a confocal microscope (Nikon A1, Tokyo, Japan, six to ten sections were imaged on confocal microscope with the resulting Z-stacks of the outflow region merged into a maximum intensity projection within the NIS-elements software. Then the images were analyzed using ImageJ software. For cell morphology analysis, cells of each group were manually analyzed with ImageJ to determine the cell morphologies, which were characterized by cell area and circularity (4π(area/perimeter^2^)) (Zhang et al., 2015). For nuclear to cytoplasmic YAP ratio analysis, we followed previously reported literature protocols (La et al., 2022). In brief, nuclear outlines were delineated in the DAPI channel using the ROI manager tool, and both the total cellular YAP intensity and the nuclear integrated fluorescence intensity were measured per cell. The YAP expression in the cytoplasm was calculated as the difference between the total cellular YAP intensity and the nuclear YAP intensity. The YAP nuclear/cytoplasmic ratio was defined as the ratio of nuclear to cytoplasmic YAP fluorescence intensity. The area and number of focal adhesions were calculated based on the previously reported literature (Güler et al., 2021). The percentage of Ki67 positive cells was calculated by the number of Ki67 positive cells to the number of DAPI positive cells per image. The percentage of nuclear β-catenin-positive cells was calculated by counting nuclear β-catenin-stained cells and total cells (DAPI) in randomly selected microscope fields (Fonseca et al., 2015).

### 2.5 Tissue immunofluorescence and analysis

Tissues from patients or mice were fixed, embedded in paraffin, and sectioned at 5 μm thickness. Sections were dewaxed, rehydrated, subjected to antigen retrieval, permeabilized, and blocked. Sections were then incubated overnight at 4 °C with primary antibodies against podoplanin (ab10288, 1:100, Abcam; ab11936, 1:200, Abcam), FAT1 (ab190242, 1:100, Abcam). After washing, appropriate fluorescent secondary antibodies were applied for 1 h at room temperature. Finally, the sections were mounted with an antifade medium containing DAPI (Abcam) and imaged using a confocal microscope (Nikon, Japan), Images were acquired using a confocal microscope (Nikon A1, Tokyo, Japan), six to ten sections were imaged on confocal microscope with the resulting Z-stacks of the outflow region merged into a maximum intensity projection within the NIS-elements software. Images were analyzed using ImageJ software. Lymphatic vessel density (LVD) was defined as the ratio of total podoplanin-positive lymphatic vessel area to total tissue area (Burchill et al., 2021). Based on previously established methods (Bhakuni et al., 2024), the mean FAT1 fluorescence intensity was calculated by dividing the FAT1 intensity by the total podoplanin-positive area. Subsequently, these values were normalized to the control group and subjected to statistical analysis.

### 2.6 siRNA transfection

The small interference RNA (siRNA) constructs targeting mouse FAT1 expression were designed and synthesized by Sangon biotech company (Shanghai, China), and the transfection was performed with Ribo Transfection Reagent (GuangZhou, China) according to the manufacturer’s protocol. The siRNA sequences were listed as follows: Mouse FAT1 siRNA (5′-UACUUGGUGACUUCGGUCC-3′).

### 2.7 Cell viability assay

Cell viability was assessed using the EdU assay. The Click-iT EdU-488 Kit (G1601, Servicebio) was used according to the manufacturer’s instructions to detect cellular proliferation. EdU-stained cells were imaged under an inverted microscope (Olympus) equipped with a digital camera (Canon). Images were analyzed using ImageJ software.

### 2.8 Spheroid assay

LEC spheroids were made as previously described (Heiss et al., 2015). Briefly, 3 × 10^3^ LECs per well were suspended in the appropriate culture medium containing 0.375% (w/v) methylcellulose (M0512, Sigma) and seeded into nonadherent round-bottom 96-well plates (3,422, Corning). Spheroids were cultured overnight. Following formation, the spheroids were transferred onto collagen-coated PA gel plates and allowed to attach. Subsequently, 300 μL of 1 mg/mL collagen I (354,249, Corning) was overlaid to cover the cells on each coverslip. Spheroids were visualized using an inverted microscope (Olympus) equipped with a digital camera (Canon).

### 2.9 Wound healing assay

Inserts (Ibidi Culture-Insert, Germany) were placed in 24-well plates coated with PA hydrogels of varying stiffness as previously reported (Wang Z. et al., 2025). Cell suspensions (5 × 10^4^ cells in 70 μL of culture medium) were added to both sides of each insert. Once the cells reached confluence, the inserts were removed, and the wells were washed twice with PBS to remove cellular debris. Cells were then cultured in medium containing 0.1% FBS. Monolayer wound closure was imaged at the indicated time points using an inverted microscope (Olympus) equipped with a digital camera (Canon). Scratch areas were quantified using ImageJ software. Wound closure rates were calculated as follows: (initial wound area–wound area at the indicated time) divided by the initial wound area.

### 2.10 RNA sequencing (RNA-seq)

SVEC4-10 cells were seeded at a density of 5 × 10^4^ cells per well onto soft (1.5 kPa) or stiff (10 kPa) hydrogels in 24-well plates (n = 3 biological replicates per condition). After 48 h, cells were harvested and lysed using Trizol. Total RNA was extracted and sequenced by Xu Ran Biological (Shanghai, China). Differentially expressed genes (DEGs) were identified based on a fold change >1.3 and p value <0.05.

### 2.11 Quantitative real-time PCR

Total RNA was extracted from cells cultured on PA gels with different stiffness using Trizol reagent (Takara, Dalian, China). RNA was reverse transcribed into complementary DNA using a cDNA Synthesis Kit (Takara, Dalian, China). Quantitative real-time PCR was performed in triplicate using a SYBR Green PCR Kit (Takara, Dalian, China) on an ABI 7500 system. Glyceraldehyde-3-phosphate dehydrogenase (GAPDH) served as the internal control. The primer sequences used are listed in Supplementary Table S1.

### 2.12 Western blotting

Cells were washed with cold PBS and lysed in RIPA lysis buffer. Then proteins were separated via sodium dodecyl sulfate-polyacrylamidegel electrophoresis (7.5%) gels and transferred to polyvinylidene difluoride membranes. The primary antibodies used in the study were as follows: anti-FAT1 (ab190242, 1:1,000, Abcam), We used β-Tubulin (10068-1-AP, 1:2000, Proteintech) as internal controls. The membranes were incubated with anti-mouse (M21001S, Abmart, Shanghai, China) or anti-rabbit (M21002S, Abmart, Shanghai, China) secondary antibodies for 1 h. Immunoblotting was visualized using an enhanced chemiluminescence solution (Pierce, Pierce, MO, United States), and the ImageQuant LAS 4000 mini (General Electric Company, Boston, MA, United States) was used to detect protein expression.

### 2.13 Statistical analysis

All statistical analyses were performed using GraphPad Prism software (GraphPad Software Inc., San Diego, CA, United States). Statistical significance between two groups was determined using Student’s t-test and one-way analysis of variance (ANOVA) was used for multiple comparations. A p-value <0.05 was considered statistically significant. At least three independent experiments were performed for each assay.

## 3 Results

### 3.1 Matrix stiffness promotes LEC morphological change and YAP nuclear localization

**FIGURE 1:**
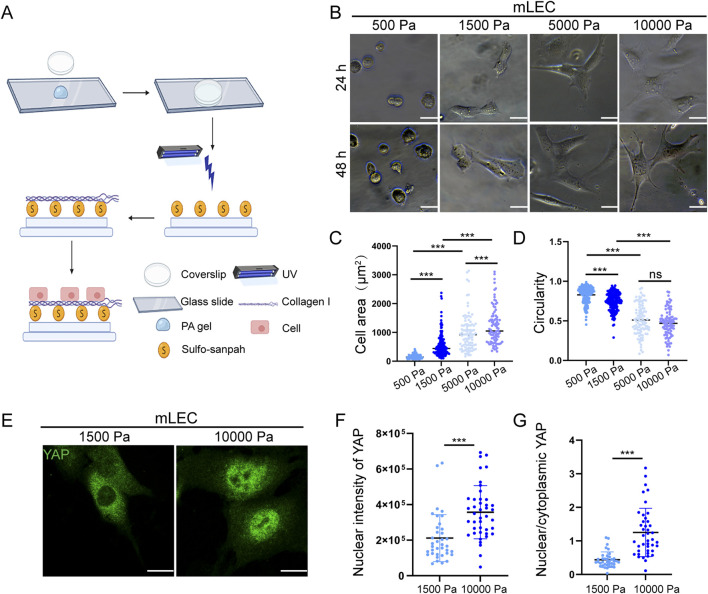
Matrix stiffness promotes LEC morphological change and YAP nuclear localization. **(A)** Schematic diagram of PA hydrogel preparation. **(B)** Representative images of mLECs cultured on hydrogels of different stiffness for 24 h and 48 h **(C,D)** Quantification of mLEC area **(C)** and circularity **(D)** after 48 h of culture on hydrogels of varying stiffness. Data are from three independent experiments (n = 229 cells for 500 Pa, n = 174 cells for 1,500 Pa, n = 96 cells for 5,000 Pa, n = 91 cells for 10,000 Pa). **(E–G)** Representative immunofluorescence images of YAP staining **(E)** and quantitative analysis of nuclear YAP fluorescence intensity **(F)** and the nuclear-to-cytoplasmic ratio of YAP **(G)** in mLECs cultured on hydrogels of different stiffness for 48 h. Data are from 6 biologically independent replicates (n = 37 cells for 1,500 Pa, n = 42 cells for 10,000 Pa). Data are presented as mean ± SD. *p < 0.05, **p < 0.01, ***p < 0.001. Scale bar: 20 μm.

In accordance with previous reports demonstrating that solid tumors display markedly increased matrix stiffness relative to normal tissues (Bordeleau et al., 2016), we used a tunable PA hydrogel system to examine the effects of tumor-associated ECM stiffness on LEC biology. As shown in Figure 1A, the PA hydrogel model that offers excellent biocompatibility and mechanical stability, is an ideal platform for matrix stiffness studies (Caliari and Burdick, 2016). Following established protocols, we generated hydrogels with precisely controlled stiffness by varying the concentrations of acrylamide and bis-acrylamide crosslinker. Based on previous reports (Filipe et al., 2024), we defined 1,500 Pa as representing normal tissue stiffness and 10,000 Pa as mimicking the stiffer tumor microenvironment, thereby creating a biologically relevant system to investigate LECs response to pathological matrix stiffness. As shown in Figures 1B–D and Supplementary Figure S1A-C, we observed that both SVEC 4–10 cells (mLECs) and human primary lymphatic endothelial cells (hLECs) exhibited distinct stiffness-dependent morphological alterations, including progressively increased cell area and significantly reduced cell circularity. These morphological changes reflected a complete phenotypic transition from the characteristic cobblestone morphology observed under physiological stiffness to an elongated, spindle-shaped appearance under tumor-like stiffness conditions. To confirm that the observed morphological alterations were specifically mediated by matrix stiffness-induced mechanotransduction in LECs, we examined the subcellular localization of Yes-associated protein (YAP), a canonical mechanosensitive transcriptional regulator (Dupont et al., 2011). As demonstrated in Figures 1E–G and Supplementary Figure S1D, E, tumor-associated matrix stiffness promoted nuclear translocation of YAP in both mLECs and hLECs, further indicating that tumor-associated ECM stiffness can functionally regulate LEC phenotype.

### 3.2 Matrix stiffness promotes lymphatic endothelial cell proliferation and migration

**FIGURE 2:**
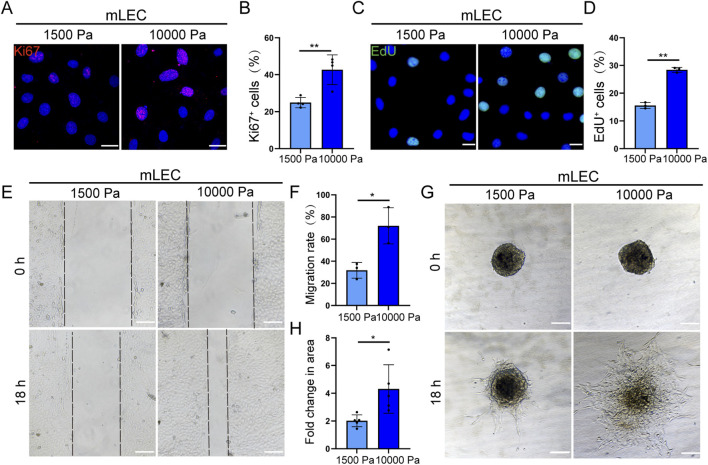
Matrix stiffness promotes lymphatic endothelial cell proliferation and migration. **(A,B)** Representative images of Ki67 immunofluorescence staining **(A)** and quantification of the percentage of Ki67-positive mLECs **(B)** cultured on hydrogels with different stiffness for 48 h (n = 4 biologically independent replicates). Scale bar: 20 μm. **(C,D)** Representative images of EdU staining **(C)** and quantification of the percentage of EdU-positive mLECs **(D)** cultured on hydrogels with different stiffness for 48 h (n = 3 biologically independent replicates). Scale bar: 20 μm. **(E,F)** Representative images of scratch assays **(E)** and quantitative analysis of mLEC migration **(F)** cultured on hydrogels with different stiffness (n = 3 biologically independent replicates). Scale bar: 100 μm. **(G,H)** Representative images of spheroid **(G)** and quantitative analysis of spheroid area by mLECs **(H)** on type I collagen-coated hydrogels with different stiffness. Data are from 3 biologically independent replicates (n = 5 spheroids). Scale bar: 100 μm. Data are presented as mean ± SD. *p < 0.05, **p < 0.01, ***p < 0.001.

To further investigate the mechanoregulatory effects of matrix stiffening on LECs function, we initially evaluated LECs proliferative capacity using Ki67 immunostaining and EdU incorporation assays. Quantitative analysis demonstrated a significant increase in the proportion of Ki67-positive and EdU-positive mLECs under high-stiffness conditions (Figures 2A–D). A similar trend of enhanced proliferation was observed in hLECs cultured on stiff substrates (Supplementary Figure S1F, G), collectively indicating that tumor-mimetic matrix stiffness promotes the proliferation of LECs.

Subsequently, a wound healing assay was conducted to evaluate the effect of tumor-associated matrix stiffness on LEC migration. The results demonstrated that high-stiffness conditions significantly enhanced the migratory potential of mLECs (Figures 2E,F). This 2D observation was further validated using a 2.5D spheroid outgrowth model, where mLECs exhibited significantly increased spreading areas on tumor-mimetic stiffness versus physiological stiffness (Figures 2G,H). These consistent findings indicate that tumor-associated matrix stiffening serves as a potent biophysical cue that enhances LEC migratory capacity through mechanotransduction pathways.

### 3.3 RNA-seq identifies FAT1 as a mechanosensitive gene in LECs

**FIGURE 3:**
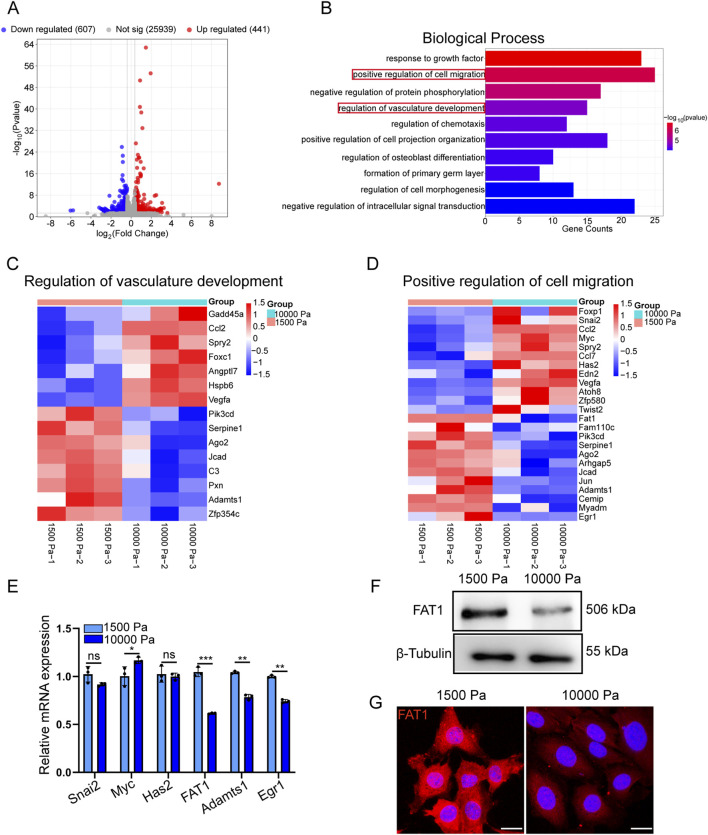
RNA-seq identifies FAT1 as a mechanosensetive gene in LECs. **(A)** Volcano plot of log2 fold change versus–log10 p-value showing transcriptional differences between mLECs cultured on 1,500 Pa and 10,000 Pa hydrogels. Vertical lines represent the 1.3-fold change cut-off, and the horizontal line indicates the p-value cut-off of 0.05. Upregulated and downregulated genes are highlighted in red and blue, respectively. **(B)** Gene enrichment analysis showing the top ten biological processes most significantly enriched between mLECs cultured on 1,500 Pa and 10,000 Pa hydrogels. **(C)** Heatmap showing the expression of genes enriched in the regulation of vascular development process. **(D)** Heatmap showing the expression of genes enriched in the positive regulation of cell migration process. **(E)** RT-qPCR validation of selected genes, with GAPDH as the housekeeping gene (n = 3 technical replicates). **(F)** Western blot analysis of FAT1 expression in mLEC protein samples cultured on hydrogels with different stiffness. **(G)** Representative immunofluorescence images of FAT1 staining in mLECs cultured on hydrogels with different stiffness for 48 h. Scale bar: 20 μm. Data are presented as mean ± SD. *p < 0.05, **p < 0.01, ***p < 0.001.

To elucidate the specific mechanisms by which elevated matrix stiffness affects the biological function of LECs, we conducted comparative RNA-seq analysis of mLECs cultured on physiologically soft (1.5 kPa) versus tumor-mimetic stiff (10 kPa) hydrogels to delineate stiffness-dependent transcriptional alterations. As shown in Figure 3A, differential expression analysis (fold change >1.3, p < 0.05) revealed that mLECs exposed to tumor-mimetic matrix stiffness exhibited 441 upregulated genes and 607 downregulated genes compared to the physiological stiffness group. Functional annotation analysis demonstrated that significant enrichment of these DEG in pathways related to positive regulation of cell migration and vasculature development (Figure 3B). Cluster heatmaps in Figures 3C,D illustrate the expression patterns of representative genes involved in these pathways.

Based on established literature (Hultgren et al., 2020; Zuo et al., 2023; Nukala et al., 2023; Li et al., 2023; Inagaki et al., 2014; Fahmy et al., 2003), we identified six candidate genes involved in endothelial cell proliferation and migration for quantitative PCR (qPCR) validation (Figure 3E). Of particular significance, FAT1, a prototypical cadherin superfamily member, exhibited the most pronounced transcriptional downregulation in mLECs grown on tumor-mimetic stiff substrates (10 kPa) relative to physiological soft matrices (1.5 kPa), with subsequent confirmation at the protein level in both mLECs and hLECs (Figures 3F,G; Supplementary Figure S1H, I).

Interestingly, we similarly observed that tumor-mimetic stiffness modulated the phenotype and function of blood endothelial cells (BECs); however, immunofluorescent staining detected no significant alteration in FAT1 expression in BECs cultured on substrates of different stiffnesses (Supplementary Figure S2A-K). Furthermore, analysis of a public dataset demonstrated that substrate stiffness did not significantly affect FAT1 expression in vascular endothelial cells (Supplementary Figure S2L). Together, these findings suggest that FAT1 may function as a lymphatic-specific gene regulated by substrate stiffness.

### 3.4 Matrix stiffness modulates the proliferative and migratory capacities of LECs through FAT1-mediated mechanotransduction

**FIGURE 4:**
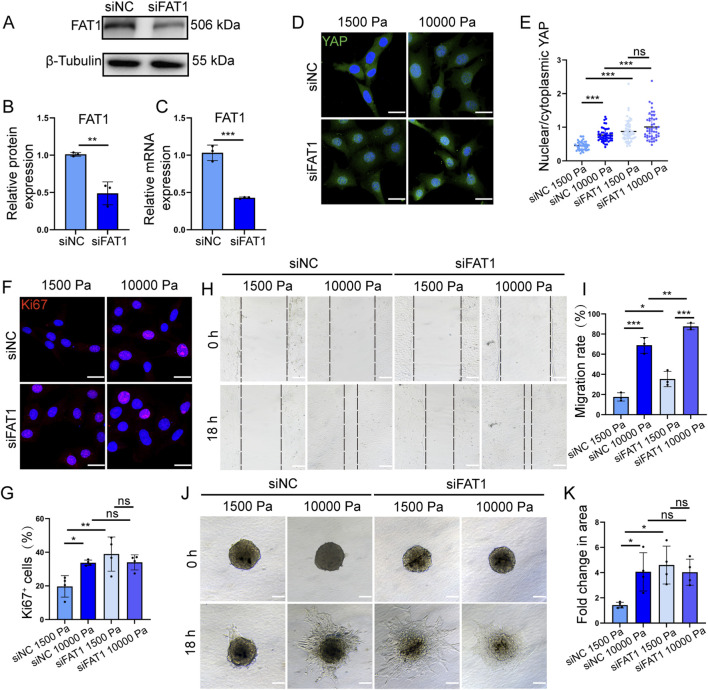
Matrix stiffness modulates the proliferative and migratory capacities of LECs through FAT1-mediated mechanotransduction. **(A)** Western blot analysis of FAT1 knockdown efficiency in mLECs. **(B)** Quantitative analysis of FAT1 protein level knockdown efficiency in mLECs (n = 3 biologically independent replicates). **(C)** Quantitative analysis of FAT1 mRNA level knockdown efficiency in mLECs (n = 3 technical replicates). **(D,E)** Representative images of YAP immunofluorescence staining **(D)** and quantitative analysis of YAP nuclear-to-cytoplasmic ratio in FAT1-knockdown mLECs **(E)** cultured on hydrogels with different stiffness for 48 h. Data are from 5 biologically independent replicates (n = 43 cells for siNC 1,500 Pa, n = 48 cells for siNC 10000 Pa, n = 57 cells for siFAT1 1,500 Pa, n = 47 cells for siFAT1 10,000 Pa). Scale bar: 20 μm. **(F,G)** Representative images of Ki67 immunofluorescence staining **(F)** and quantitative analysis of the percentage of Ki67-positive cells in FAT1-knockdown mLECs **(G)** cultured on hydrogels with different stiffness for 48 h (n = 4 biologically independent replicates). Scale bar: 20 μm. **(H,I)** Representative images of scratch assays **(H)** and quantitative analysis of FAT1-knockdown mLECs migration **(I)** cultured on hydrogels with different stiffness (n = 3 biologically independent replicates). Scale bar: 100 μm. **(J,K)** Representative images of spheroids **(J)** and quantitative analysis of spheroid area of FAT1-knockdown mLECs **(K)** pre-cultured in low-attachment plates and then seeded on type I collagen-coated hydrogels with different stiffness. Data are from 3 biologically independent replicates (n = 4 spheroids). Scale bar: 100 μm. Data are presented as mean ± SD. *p < 0.05, **p < 0.01, ***p < 0.001.

To further validate FAT1’s role as a mechanosensitive regulator in LEC function, we achieved efficient FAT1 knockdown in SVEC4-10 cells using small interfering RNA (siRNA), with significant reduction at both the mRNA and protein levels (Figures 4A–C). FAT1-silenced and negative control (NC) mLECs were subsequently cultured on hydrogels mimicking physiological or tumor-associated stiffness. Immunofluorescence analysis of YAP subcellular localization (Figures 4D,E) demonstrated that FAT1 knockdown significantly increased the nuclear-to-cytoplasmic ratio of YAP in mLECs grown on both soft and stiff matrices compared to NC groups, suggesting that FAT1 loss potentiates YAP-mediated mechanotransduction pathways.

Our further investigations revealed that FAT1 knockdown significantly increased the proliferative activity of mLECs grown on matrices of physiological stiffness (Figures 4F,G), though no synergistic effect was observed between FAT1 silencing and matrix stiffening in promoting cell proliferation.

We next evaluated the consequences of FAT1 knockdown on mLEC migratory behavior. 2D migration assays demonstrated consistent enhancement across different mechanical environments, with FAT1 knockdown mLECs showing improved motility on both matrices with physiological stiffness and tumor-mimetic stiffness (Figures 4H,I). This mechanosensitive phenotype was further confirmed using 2.5D spheroid outgrowth assays. FAT1 knockdown elevated the migratory ability of mLEC spheroids on soft matrices to levels comparable to those of the rigid matrix control group (Figures 4J,K), quantitative analysis of spheroid spreading areas confirmed the absence of additive effects between FAT1 silencing and matrix stiffening.

Together, these results establish FAT1 as a possible signaling molecule important to the regulation of LEC mechanotransduction, affecting both proliferative and migratory responses to extracellular matrix stiffness.

### 3.5 FAT1 deficiency promotes LEC proliferation through activation of Wnt/β-catenin signaling while simultaneously enhancing migratory capacity via focal adhesion-dependent cytoskeletal reorganization

**FIGURE 5:**
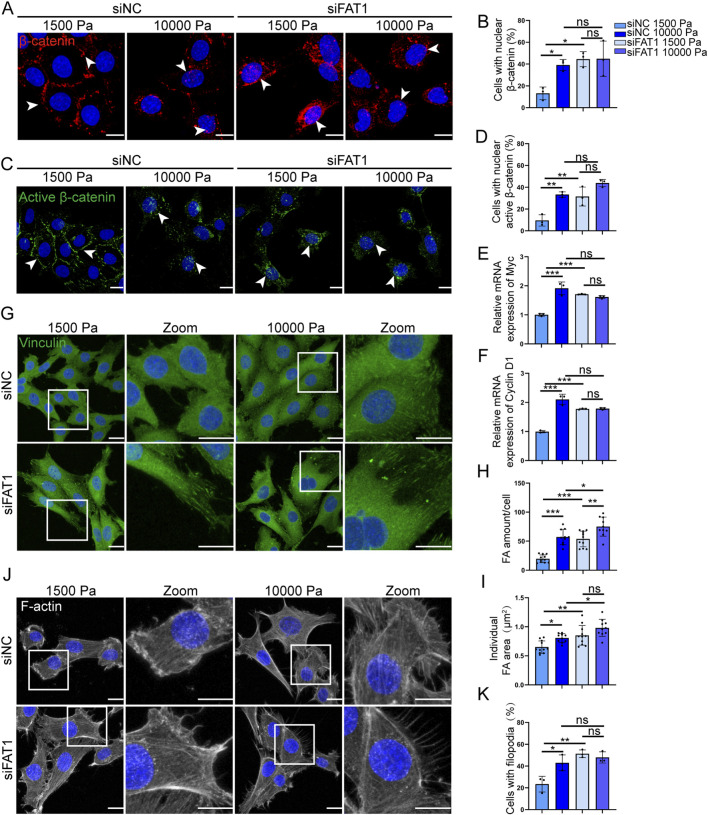
FAT1 deficiency promotes LEC proliferation through activation of Wnt/β-catenin signaling while simultaneously enhancing migratory capacity via focal adhesion-dependent cytoskeletal reorganization. **(A,B)** Representative images of total β-catenin staining **(A)** and quantitative analysis of the percentage of nuclear total β-catenin-positive cells in FAT1-knockdown mLECs **(B)** cultured on hydrogels with different stiffness for 48 h (n = 3 biologically independent replicates). Scale bar: 20 μm. **(C,D)** Representative images of active β-catenin staining **(C)** and quantitative analysis of the percentage of nuclear active β-catenin-positive cells in FAT1-knockdown mLECs **(D)** cultured on hydrogels with different stiffness for 48 h (n = 3 biologically independent replicates). Scale bar: 20 μm. **(E)** RT-qPCR analysis of the mRNA expression of Wnt target gene Myc in mLECs cultured on hydrogels with different stiffness for 48 h (n = 3 technical replicates). **(F)** RT-qPCR analysis of the mRNA expression of Wnt target gene Cyclin D1 in FAT1-knockdown mLECs cultured on hydrogels with different stiffnesses for 48 h (n = 3 technical replicates). **(G)** Representative images of vinculin staining in FAT1-knockdown mLECs cultured on hydrogels with different stiffness for 48 h. Scale bar: 20 μm. **(H)** Quantitative analysis of the number of FA per FAT1-knockdown mLEC cultured on hydrogels with different stiffness for 48 h. Data are from 3 biologically independent replicates (n = 11 cells for siNC 1,500 Pa, n = 10 cells for siNC 10000 Pa, n = 11 cells for siFAT1 1,500 Pa, n = 10 cells for siFAT1 10,000 Pa). **(I)** Quantitative analysis of individual FA area in FAT1-knockdown mLECs cultured on hydrogels with different stiffness for 48 h. Data are from 3 biologically independent replicates (n = 11 cells for siNC 1,500 Pa, n = 10 cells for siNC 10000 Pa, n = 11 cells for siFAT1 1,500 Pa, n = 10 cells for siFAT1 10,000 Pa). **(J,K)** Representative images of F-actin staining **(J)** and quantitative analysis of the percentage of cells with filopodia in FAT1-knockdown mLECs **(K)** cultured on hydrogels with different stiffness for 48 h (n = 3 biologically independent replicates). Scale bar: 20 μm. The colors scheme in the bar graphs (Figures 5D–F,H,I,K) correspond to the color scheme defined in Figure 5B. Data are presented as mean ± SD. *p < 0.05, **p < 0.01, ***p < 0.001.

Given Wnt/β-catenin’s crucial role in proliferation, we investigated its involvement in FAT1-regulated LEC proliferation. Immunofluorescence analysis revealed that both total and non-phosphorylated (active) β-catenin shifted from membrane localization (physiological stiffness) to nuclear/perinuclear accumulation (tumor-associated stiffness) in mLECs (Figures 5A–D). Notably, FAT1 knockdown mimicked this translocation even at physiological stiffness, indicating FAT1 normally suppresses Wnt signaling in compliant environments. QPCR confirmed Wnt activation, showing increased expression of downstream targets Myc and Cyclin D1 after either matrix stiffening or FAT1 depletion (Figures 5E,F), further demonstrating FAT1 mechanosensitively regulates LEC proliferation via Wnt/β-catenin signaling.

As vinculin serves as the central mechanotransducer coordinating migration through focal adhesion (FA) dynamics (Holland et al., 2024), we examined FAT1’s regulation of LEC migration via vinculin. Immunofluorescence analysis (Figure 5G) revealed that FAT1 knockdown increased both the number and area of FAs in mLECs on matrices with physiological stiffness, reaching levels comparable to those observed in the NC group cells grown on tumor-mimetic stiff matrices. Furthermore, under tumor-mimetic stiffness conditions, FAT1 silencing further enhanced focal adhesion formation, increasing both their quantity (Figure 5H) and size (Figure 5I). These findings suggest that FAT1 normally suppresses FA formation to modulate mechanical responsiveness.

Since cells sense extracellular mechanical cues by connecting the cytoskeleton to FAs (Wang et al., 2024), we further elucidated FAT1’s regulatory role in LEC cytoskeletal remodeling. Immunofluorescence analysis revealed a significant increase in filopodia-positive mLECs cultured on matrices with tumor-like stiffness compared to matrices with physiological stiffness (Figure 5J). Strikingly, genetic ablation of FAT1 recapitulated this pro-migratory cytoskeletal phenotype even in compliant microenvironments, suggesting that FAT1 suppresses LEC migratory potential through negative regulation of filopodia formation (Figure 5K).

### 3.6 Reduced FAT1 expression in tumor-associated lymphatic vessels correlates with increased lymphatic vessel density

**FIGURE 6:**
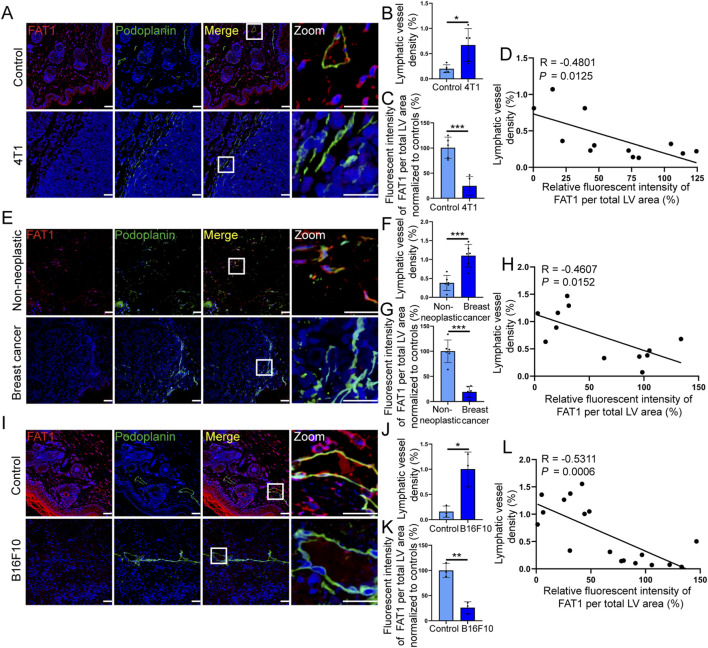
Reduced FAT1 expression in tumor-associated lymphatic vessels correlates with increased lymphatic vessel density. **(A)** Representative images of co-immunofluorescence staining for PDPN and FAT1 in normal mouse tissue and 4T1 tumor tissue. Scale bar: 100 μm. **(B)** Quantitative analysis of lymphatic vessel density in normal and 4T1 tumor-bearing mouse tissues (n = 5 mice per group). **(C)** Quantitative analysis of FAT1 fluorescence intensity per total lymphatic vessel (LV) area in normal and 4T1 tumor-bearing mouse tissues (n = 5 mice per group). **(D)** Correlation analysis between relative FAT1 fluorescence intensity and lymphatic vessel density in normal and 4T1 tumor-bearing mouse tissues (n = 12 fields of view from 10 mice). **(E)** Representative images of co-immunofluorescence staining for PDPN and FAT1 in human non-neoplastic and breast cancer tissues. Scale bar: 100 μm. **(F)** Quantitative analysis of lymphatic vessel density in human non-neoplastic and breast cancer tissues (n = 6 human tissues). **(G)** Quantitative analysis of FAT1 fluorescence intensity per total LV area in human non-neoplastic and breast cancer tissues (n = 6 human tissues). **(H)** Correlation analysis between relative FAT1 fluorescence intensity and lymphatic vessel density in human non-neoplastic and breast cancer tissues (n = 12 human tissues). **(I)** Representative images of co-immunofluorescence staining for PDPN and FAT1 in normal mouse tissue and B16F10 tumor tissue. Scale bar: 100 μm. **(J)** Quantitative analysis of lymphatic vessel density in normal and B16F10 tumor-bearing mouse tissues (n = 3 mice per group). **(K)** Quantitative analysis of FAT1 fluorescence intensity per total LV area in normal and B16F10 tumor-bearing mouse tissues (n = 3 mice per group). **(L)** Correlation analysis between relative FAT1 fluorescence intensity per total LV area and lymphatic vessel density in normal and B16F10 tumor-bearing mouse tissues (n = 18 fields of view from 6 mice). Data are presented as mean ± SD. *p < 0.05, **p < 0.01, ***p < 0.001.

Building upon our *in vitro* findings that tumor-mimetic extracellular matrix stiffness suppresses FAT1 expression in LECs, we next characterized the expression pattern of FAT1 in lymphatic vessels during tumor progression *in vivo*. In the murine 4T1 breast cancer model, immunofluorescence co-staining with the lymphatic-specific marker podoplanin (PDPN) and FAT1 revealed a significant reduction in FAT1 fluorescence intensity within tumor-associated lymphatic vessels compared to those in normal mammary tissue (Figures 6A,C). Moreover, we observed an increase in lymphatic vessel density (LVD) (Figure 6B). Subsequent correlation analysis revealed a significant negative correlation between LVD and relative FAT1 fluorescence intensity, suggesting that reduced FAT1 expression in tumor-associated lymphatic vessels is associated with higher LVD (Figure 6D).

Consistent with these preclinical findings, analysis of matched clinical specimens from human breast cancer patients indicated that a negative correlation between FAT1 expression and lymphatic vessel density was also observed in human tumor-associated lymphatic vessels, supporting the existence of this relationship in clinical samples (Figures 6E–H). To establish the broader relevance of this phenomenon, we extended our investigation to the B16F10 melanoma model, in which a similar negative correlation between FAT1 expression and lymphatic vessel density was also observed in tumor-associated lymphatic vessels and normal lymphatic vessels (Figures 6I–L).

Collectively, our results suggest that matrix mechanotransduction may serve as a key regulatory mechanism modulating tumor-associated lymphatic function through the regulation of FAT1 expression.

## 4 Discussion

The stiffness of the tumor extracellular matrix often increases as a result of enhanced crosslinking and elevated matrix density, substantially affecting cellular behavior and fate (Cambria et al., 2024). Consistent with prior reports demonstrating that matrix stiffening within the tumor microenvironment induces pathological phenotypes in vascular endothelial cells—characterized by aberrant proliferation, migration, cytoskeletal remodeling, and compromised vascular function (Bordeleau et al., 2016; Romero-López et al., 2017). Our findings establish that LECs exhibit pronounced mechanosensitivity. Specifically, tumor-mimetic matrix stiffness significantly enhances LEC proliferation and migratory capacity.

**FIGURE 7:**
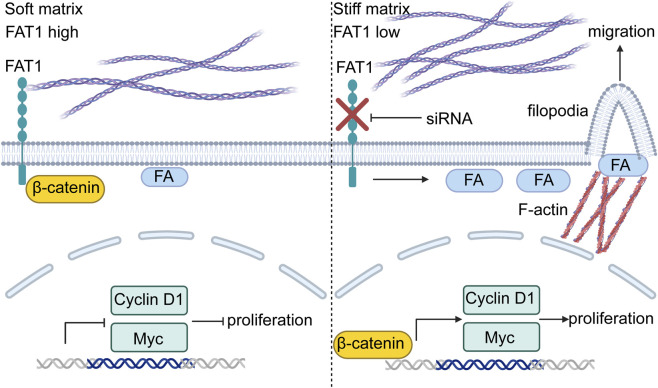
Proposed schematic diagram of matrix stiffness regulation on LEC proliferation and migration via FAT1.

To elucidate the mechanism by which substrate stiffness modulates LEC behavior, we performed RNA-Seq analysis and identified FAT1 as a putative mechanosensitive regulator. FAT1—encoding a protocadherin family member—ranks among the most frequently mutated genes in human cancers (Cao et al., 2024). Although its roles in tumor cells and vascular smooth muscle cells are established (Cao et al., 2016; Pastushenko et al., 2020), FAT1 function in lymphatic endothelial cells remains largely unexplored. Remarkably, recent studies indicate that vascular endothelial FAT1 suppresses YAP/TAZ signaling, thereby inhibiting endothelial proliferation and angiogenesis (Li et al., 2023). Here, we provide the first evidence that FAT1 may act as a mechanosensitive molecule in LECs. We demonstrate that tumor-mimetic matrix stiffness significantly downregulates FAT1 expression, which likely contributing to enhanced proliferative and migratory capacities in LECs. Notably, FAT1 knockdown on matrices with physiological stiffness recapitulated many of the aberrant phenotypes observed on stiff matrices, suggesting that FAT1 loss is both necessary and sufficient to induce these cellular responses.

Mechanistically, we observed that knockdown of FAT1 was sufficient to induce increased nuclear translocation of YAP on both soft and stiff matrices compared to the NC group. This finding is consistent with prior reports in cancer cells (Pastushenko et al., 2020) and provides further mechanistic insight into the altered biological functions of mLECs on stiff matrices relative to soft matrices. Given the critical role of Wnt/β-catenin signaling in regulating cell proliferation—a key functional alteration observed in FAT1-deficient cancer cells, we next examined whether this pathway is activated upon FAT1 loss in mLECs. We observed that FAT1 knockdown promoted the activation of β-catenin in the Wnt pathway and upregulation of key downstream target genes, thereby enhancing the proliferation of lymphatic endothelial cells, extending Morris et al.'s observations in tumor cells (Morris et al., 2013) to the lymphatic endothelium. Notably, siRNA-mediated FAT1 knockdown achieved comparable reduction levels to those induced by matrix stiffening. Furthermore, we unexpectedly observed that FAT1 knockdown increased the number and area of focal adhesions in mLECs. Intriguingly, even mLECs cultured on physiological soft matrices with FAT1 knockdown demonstrated FA numbers comparable to or exceeding those NC LECs on tumor-mimetic stiff matrices. This observation aligns with recent work by Zhao et al., who showed that FAT1 silencing in triple-negative breast cancer cells led to increased FA length (Zhao et al., 2025), thus supporting the hypothesis that reduced FAT1 may enhances mechanosensitivity of LECs via modulation of focal adhesions. Given FAs function as primary mechanosensors coupling extracellular cues to cytoskeletal dynamics, we examined actin reorganization. The result shows that both substrate stiffening and FAT1 knockdown independently promoted peripheral filopodia formation. Consequently, FAT1-deficient mLECs formed abundant filopodia-like protrusions even on soft matrices. Since filopodia facilitate cell migration (Wang Y. et al., 2025), which may explain the enhanced migratory ability of FAT1-knockdown mLECs on matrices with physiological stiffness. Intriguingly, filopodia formation has been demonstrated to promote endothelial tip cell specification and sprouting (Hiepen et al., 2025), these observations could imply a potential role for FAT1 in modulating mLEC sprouting and lymphangiogenesis.

Previous studies have reported that the expression of FAT1 is downregulated in breast tumor tissues compared to normal tissues, and it is also reduced in breast cancer cells relative to normal mammary epithelial cells (Zhao et al., 2025). However, the expression pattern of FAT1 within stromal cells in the tumor microenvironment remains poorly characterized. In this study, we observed that FAT1 expression is significantly decreased in tumor-associated LECs compared to LECs in normal tissues. However, whether FAT1 regulates lymphangiogenesis *in vivo* requires further investigation.

Together, our findings demonstrate that matrix stiffening significantly promotes LEC proliferation and migration. Notably, we identify FAT1 as a mechanosensitive regulator in LECs that links matrix stiffness to these cellular behaviors (Figure 7), both closely related to lymphangiogenesis. Targeting tumor stiffness or FAT1-dependent pathways may represent promising strategies to suppress tumor-driven lymphangiogenesis and metastasis.

## Data Availability

The datasets presented in this study can be found in online repositories. The names of the repository/repositories and accession number(s) can be found below: Genome Sequence Archive (GSA) in National Genomics Data Center and accession number CRA031476 (https://ngdc.cncb.ac.cn/gsa/browse/CRA031476).

## Supplementary material

Supplementary Figure S1 The effect of substrate stiffness on human primary lymphatic endothelial cells (hLECs). **(A)** Representative images of hLECs cultured on hydrogels of different stiffness for 48 h. Scale bar: 50 μm. **(B,C)** Quantification of hLECs area **(B)** and circularity **(C)** after 48 h of culture on hydrogels with varying stiffness. Data are from three independent experiments (n = 121 cells for 1,500 Pa, n = 133 cells for 10000 Pa). **(D,E)** Representative immunofluorescence images of YAP staining **(D)** and quantitative analysis of the nuclear-to-cytoplasmic ratio of YAP in hLECs **(E)** cultured on hydrogels of different stiffness for 48 h. Data are from 3 biologically independent replicates (n = 30 cells for 1,500 Pa, n = 31 cells for 10000 Pa). Scale bar: 20 μm. **(F,G)** Representative images of Ki67 immunofluorescence staining **(F)** and quantification of the percentage of Ki67-positive hLECs **(G)** cultured on hydrogels with different stiffness for 48 h (n = 3 biologically independent replicates). Scale bar: 20 μm. **(H,I)** Representative immunofluorescence images of FAT1 staining **(H)** and quantitative analysis of FAT1 mean fluorescence intensity (MFI) in hLECs **(I)** cultured on hydrogels of different stiffness (n = 3 biologically independent replicates). Data are presented as mean ± SD. **p* < 0.05, ***p* < 0.01, ****p* < 0.001.

Supplementary Figure S2 The effect of substrate stiffness on blood endothelial cells (BEC). **(A)** Representative images of BECs cultured on hydrogels of different stiffness for 48 h. Scale bar: 50 μm. **(B,C)** Quantification of BEC area **(B)** and circularity **(C)** after 48 h of culture on hydrogels with varying stiffness. Data are from three independent experiments (n = 156 cells for 1,500 Pa, n = 175 cells for 10000 Pa). **(D,E)** Representative immunofluorescence images of YAP staining **(D)** and quantitative analysis of the nuclear-to-cytoplasmic ratio of YAP in BECs **(E)** cultured on hydrogels of different stiffness for 48 h. Data are from 3 biologically independent replicates (n = 36 cells). Scale bar: 20 μm. **(F,G)** Representative images of Ki67 immunofluorescence staining **(F)** and quantification of the percentage of Ki67-positive BECs **(G)** cultured on hydrogels with different stiffness for 48 h (n = 3 biologically independent replicates). Scale bar: 20 μm. **(H,I)** Representative images of scratch assays of BECs **(H)** and quantitative analysis of BEC migration in scratch assays **(I)** cultured on hydrogels with different stiffness (n = 3 biologically independent replicates). Scale bar: 100 μm. **(J,K)** Representative immunofluorescence images of FAT1 staining **(J)** and quantitative analysis of FAT1 mean fluorescence intensity (MFI) in BECs **(K)** cultured on hydrogels with different stiffness for 48 h (n = 3 biologically independent replicates). Scale bar: 20 μm. **(L)** Quantitative analysis of FAT1 transcriptional levels in human pulmonary artery endothelial cells (HPAECs) cultured on matrices of different stiffness. Soft: HPAECs cultured on a soft matrix (1,000 Pa); Stiff: HPAECs cultured on a stiff matrix (50000 Pa) (Dataset source: GSE75793). Data are presented as mean ± SD. **p* < 0.05, ***p* < 0.01, ****p* < 0.001.
