# Supplementary figures and images for: Extracellular matrix stiffness regulates the proliferation and migration capacities of lymphatic endothelial cells via FAT1
Source: Front Cell Dev Biol. 2025 Oct 27;13:1667154. doi: 10.3389/fcell.2025.1667154 (PMC12604528; doi:10.3389/fcell.2025.1667154)

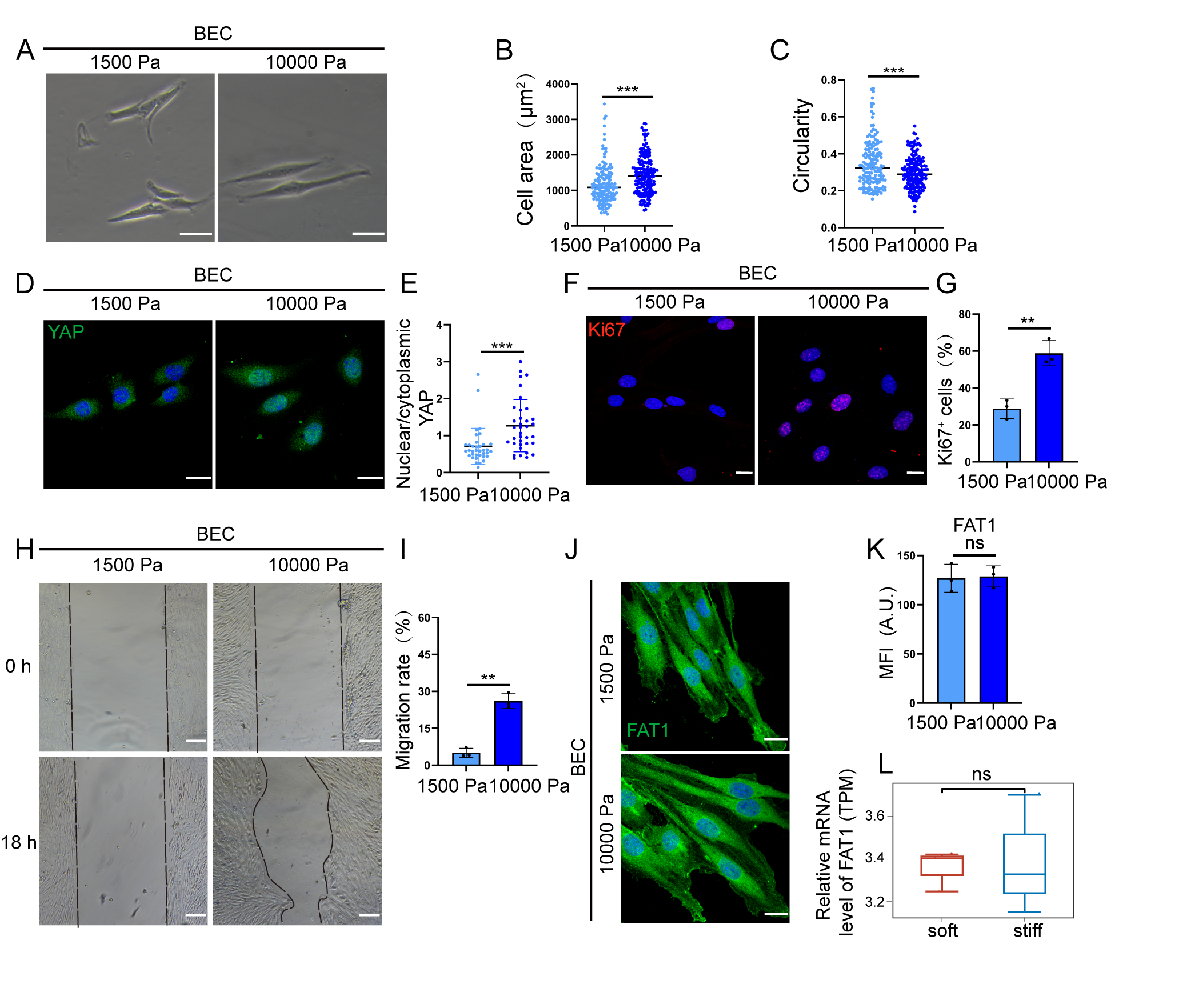

Supplement: Supplementary file 1 [file Image2.tif]

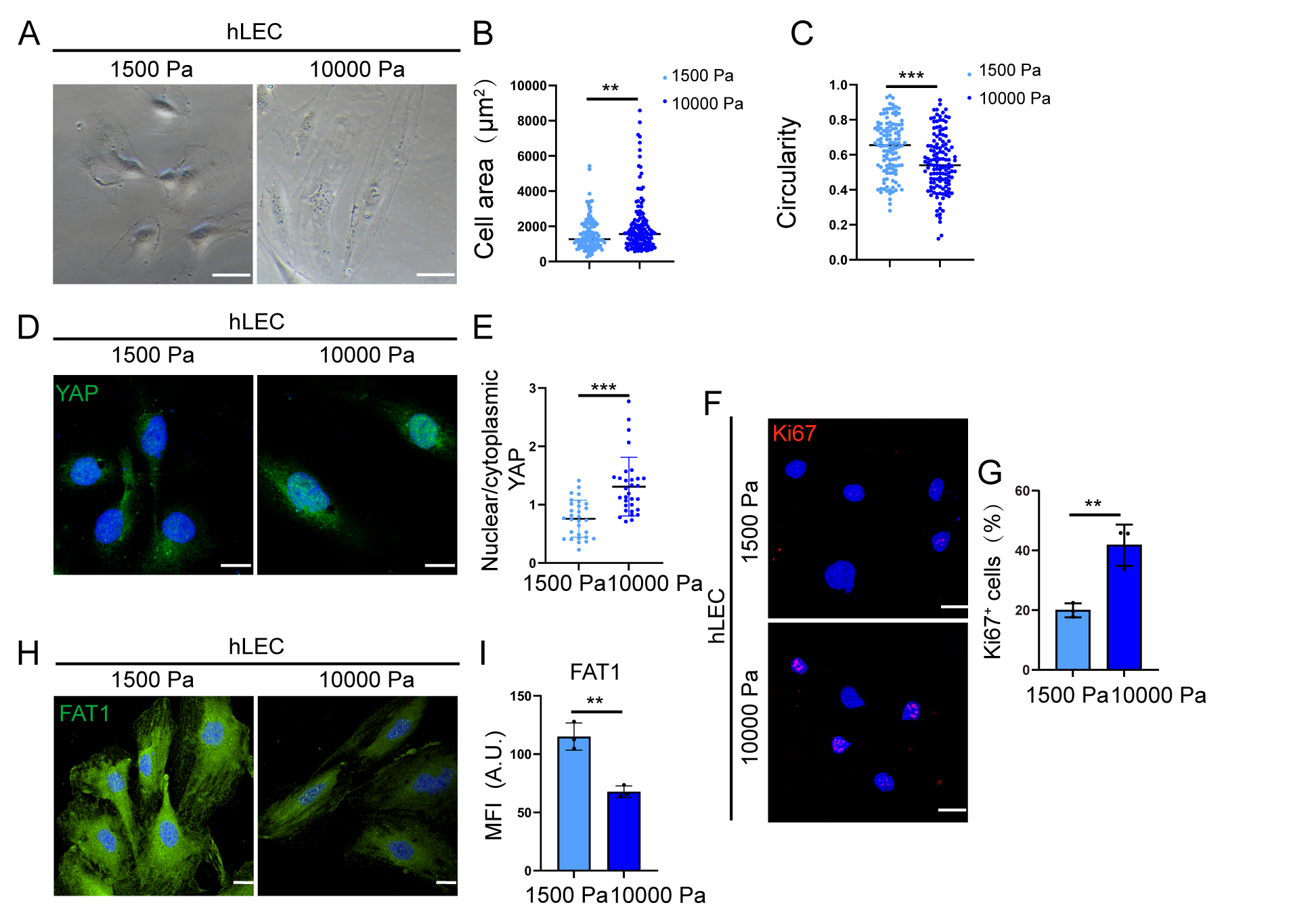

Supplement: Supplementary file 2 [file Image1.tif]
